# Supplementary material for: New Insights in the Occurrence of Venous Thromboembolism in Critically Ill Patients with COVID-19—A Large Postmortem and Clinical Analysis
Source: Viruses. 2022 Apr 14;14(4):811. doi: 10.3390/v14040811 (PMC9027529; doi:10.3390/v14040811)
Supplement: Supplementary file 1 [file viruses-14-00811-s001.zip › viruses-1662004-supplementary.pdf]

Supplementary Table S1: Baseline characteristics of age- and sex-matched COVID-19 (n=21) and non-COVID-19 (n=21) patients from intensive care units all over the city of Hamburg, autopsied at the Institute of Legal Medicine (University Medical Center Hamburg-Eppendorf, Germany), are illustrated. Patients were included from consecutive cohorts of COVID-19 patients between January 1st, 2020, until December 31st, 2020, and non-COVID-19 patients between January 1st, 2019, until February 29th, 2020. Numbers with frequencies and median with interquartile ranges are illustrated. Abbreviations: ICU, intensive care unit; BMI, body mass index; MV, mechanical ventilation; RRT, renal replacement therapy; ECMO, extracorporeal membrane oxygenation.

	**Non-COVID-19 ICU patients**	**COVID-19 ICU patients**	**Overall patients**
	n = 21	n = 21	n = 42
	Sociodemographic variables		
Age	74.0 (57.0-81.0)	74.0 (57.0-81.0)	74.0 (57.0-81.0)
Sex			
Female	3 (14.3%)	3 (14.3%)	6 (14.3%)
Male	18 (85.7%)	18 (85.7%)	36 (85.7%)
BMI	28.0 (24.0-30.0)	28.7 (27.3-33.3)	28.3 (24.0-32.0)
	ICU related therapy		
MV	20 (95.2%)	16 (76.2%)	36 (85.7%)
RRT	7 (33.3%)	8 (38.1%)	15 (35.7%)
ECMO	1 (4.8%)	2 (9.5%)	3 (7.1%)
	Deep vein thrombosis		
Not examined	18 (85.7%)	7 (33.3%)	25 (59.5%)
0	3 (14.3%)	7 (33.3%)	10 (23.8%)
1	0 (0.0%)	7 (33.3%)	7 (16.7%)
	Pulmonary embolisms		
0	21 (100.0%)	12 (57.1%)	33 (78.6%)
1	0 (0.0%)	9 (42.9%)	9 (21.4%)
